# Identifying and understanding dietary transitions and nutrient deficiency from loyalty card digital footprints

**DOI:** 10.23889/ijpds.v8i3.2266

**Published:** 2023-09-11

**Authors:** Roberto Mansilla, Gavin Long, Simon Welham, John Harvey, Evgeniya Lukinova, Georgiana Nica-Avram, Gavin Smith, Andrew Smith, James Goulding

**Affiliations:** 1 N/LAB, Nottingham University Business School, Jubilee Campus, University of Nottingham.; 2 Division of Food, Nutrition & Dietetics, School of Biosciences, Sutton Bonington, University of Nottingham.

## Introduction & Background

The shift towards plant-based diets remains on the rise. Several observational studies have suggested that adopting these diets can result in some fundamental nutrient deficiencies, such as iodine deficiency. This can be especially harmful to a developing fetus, leading to growth impairment and, in extreme cases, cretinism. Nonetheless, understanding of long-term health consequences of this shift remains a challenge, particularly regarding nutritional impact at broader population scales.

## Objectives & Approach

Our study focuses on the effects of transitioning to plant-based diets on the purchasing and assumed intake of essential nutrients like iodine, calcium, and vitamin B12. We analysed anonymized shopping records of 10,626 loyal customers who switched from regular milk to alternative milk products. By matching the transaction data with nutritional information, we estimated the weekly nutrient purchases before and after the transition. Our data was collected from a national food retailer across the UK.

## Relevance to Digital Footprints

Loyalty-card transactional logs held by retailers reflect a valuable lens into nutritional intake data. This data can provide insight into the potential impact of purchasing behaviours, such as the potential health effects of dietary changes at scale. Our approach leverages AI modelling accompanied by rigorous variable importance methods to uncover potentially hidden insights on the impact of nutritional shifts to plant-based goods.

## Results

Results indicate that 83% of individuals deemed regular customers, who switched to plant-based milk, experienced a decrease in their purchases of iodine (44%), calcium (30%), and vitamin B12 (39%) from their normal purchase patterns at the retailer. Additionally, 57% of these individuals decreased their iodine purchases by more than 50\%. The reduction in these nutrients is even more significant for those who switch to plant-based dairy and meat products.

## Conclusions & Implications

Our research indicates that dietary changes, such as switching from purchasing regular milk to alternative milks, may lead to insufficient intake of essential dietary nutrients such as iodine. This represents a significant potential health concern for the public if not remediated, especially in countries that do not require salt to be fortified with iodine.

